# Physician’s Manual of Therapeutics

**Published:** 1901-02

**Authors:** 


### Books and Magazines.

---

PHYSICIAN'S MANUAL OF THERAPEUTICS, referring especially to the products of the Pharmaceutical and Biological Laboratories of Parke, Davis & Co. Flexible morocco; 12 mo.; pp. 526; Detroit, 1900.

In this preface to this excellent little book, the author says "There has been much well-meaning derision of 'elegant pharmacy', but we do not sympathize with it; and the products enumerated in this volume are deliberately made as sightly, palatable and inviting as possible without sacrificing any of that therapeutic efficacy, which is the supreme end of medication." That sentence contains, as in a nutshell, the whole secret of the great success of Messrs. Parke, Davis & Co. While their pharmaceutical preparations are elegant, they are honest, and always up to the required standards of medicinal strength. The Physicians' Manual has evidently been brought forth under the same conditions. It has a handsome cover, it is durably bound, and an examination of its pages discloses the fact that it is full of "meat". We admire the scientific tone which is evident in each section of the book.

Part one is entitled "Therapeutic Suggestions," and, as its title indicates, comprises various useful hints on the prophylaxis and treatment of the diseases, the names of which appear in alphabetical order, as sub-heads. A number of useful tables have been arranged to assist the inquirer in the differential diagnosis of the exanthemata, the calculation of metric values, thermometric equivalents, etc. The body of the book is given over to the department of "Materia Medica," which is in brief an alphabetic catalogue of drugs, and seems to be thoroughly complete and up-to-date. No secret preparations are mentioned, nor is mention made of antiquated or obsolete remedies. The various preparations of the different drugs, such as pills, tablets, capsules, elixirs, are alphabetically listed under their respective heads and may be found without confusion or delay. The printer and bookbinder have done their work well, and there is no doubt that the demand for this excellnt "Manual" will more than compensate the publishers for what must have been a considerable outlay of time, effort and money.

A copy of the Manual will be sent free to any reader of the TEXAS MEDICAL JOURNAL on request if the JOURNAL be mentioned. —ED.

